# Gender Differences in the *VDR-*FokI Polymorphism and Conventional Non-Genetic Risk Factors in Association with Lumbar Spine Pathologies in an Italian Case-Control Study

**DOI:** 10.3390/ijms16023722

**Published:** 2015-02-09

**Authors:** Alessandra Colombini, Marco Brayda-Bruno, Lucia Ferino, Giovanni Lombardi, Vincenzo Maione, Giuseppe Banfi, Sabina Cauci

**Affiliations:** 1 IRCCS Galeazzi Orthopaedic Institute, via R. Galeazzi 4, 20161 Milan, Italy; E-Mails: giovanni.lombardi@grupposandonato.it (G.L.); banfi.giuseppe@fondazionesanraffaele.it (G.B.); 2 Department of Orthopedics and Traumatology—Vertebral surgery III—Scoliosis, IRCCS Galeazzi Orthopaedic Institute, via R. Galeazzi 4, 2016 Milan, Italy; E-Mail: marco.brayda@spinecaregroup.it; 3 Department of Medical and Biological Sciences, University of Udine, P.le Kolbe 4, 33100 Udine, Italy; E-Mails: ferino.lucia@spes.uniud.it (L.F.); maionevincenzo@gmail.com (V.M.); sabina.cauci@uniud.it (S.C.); 4 Vita e Salute San Raffaele University, via Olgettina 58, 20132 Milan, Italy

**Keywords:** vitamin D receptor, polymorphism, lumbar spine pathologies, risk factors, gender-related differences, Italian population

## Abstract

Recently, the FokI polymorphism (rs2228570) in the vitamin D receptor gene (*VDR*) and conventional risk factors were associated with spine disorders in the Italian population, but without gender analysis. Two-hundred and sixty-seven patients (149 males, 118 females) with lumbar spine disorders were assessed by magnetic resonance imaging (MRI) and 254 (127 males, 127 females) asymptomatic controls were enrolled. The exposure to putative risk factors was evaluated and FokI polymorphism was detected by PCR-restriction fragment length polymorphism (PCR-RFLP). An association between lumbar spine pathologies and higher than average age; overweight; family history; lower leisure physical activity; smoking habit; higher number of hours/day exposure to vibration and more sedentary or intense physical job demand was observed in male patients. In contrast, in females, only higher age, overweight, family history and lower leisure physical activity were risk factors. *FF* genotype was a 2-fold risk factor to develop discopathies and/or osteochondrosis concomitant with disc herniation for both gender patients, while heterozygous *Ff* was protective for females only. In males only *ff* genotype was protective for discopathies and/or osteochondrosis and *F* allele was a 2-fold risk factor for hernia; discopathies; discopathies and/or osteochondrosis. Sex-related differences in voluntary behaviors, exposure to environmental risks and genetic background could be crucial for a gender-differentiated management of patients with spine disorders.

## 1. Introduction

Since 1998 the vitamin D receptor gene (*VDR*) has been studied as a genetic factor putatively predisposing to spine pathologies [1,2]. The C > T single nucleotide polymorphism FokI (rs10735810, merged into rs2228570) in the *VDR* sequence represents an independent polymorphic site, affecting the structure and the function of the encoded protein [3]. The allelic variants of this exonic polymorphism code for a structurally different receptor protein, from a 424 amino acids long wild-type encoded by the *F* allele (C) to a 427 amino acids long protein produced by the *f* allele (T), associated to a different efficiency of *VDR* binding with the transcription factor II B (TFIIB) [4,5]. This results in a different ability to induce transcription of vitamin D-dependent genes with the shorter wild-type protein interacting more efficiently with TFIIB and showing a higher transcriptional rate [3,6]. Based on these studies, investigations concerning the possible association of the *VDR*-FokI polymorphism with disc degeneration may be particularly interesting.

Established evidence indicates that the vitamin D endocrine system is involved in the modulation of skeletal metabolism, immunological response, proliferation and differentiation of a wide variety of cell types [7,8,9,10]. Vitamin D action mediated by its receptor appears to be selective and to vary depending on cell type [10,11]. Recent findings detected the presence of VDR in the human intervertebral disc nucleus pulposus and annulus fibrosus cells and showed that vitamin D active metabolites regulate proliferation, matrix genes expression and specific cytokine and protein production of disc cells [12,13]. These *in vitro* studies indicated that the biologic interaction of disc cells with the vitamin D metabolites may influence disc health; consequently altered vitamin D signaling could have a role in the pathophysiology of disc degeneration.

The association of the *VDR*-FokI polymorphism with lumbar spine pathologies implicating lumbar disc degeneration [2,14,15,16,17] and the influence of non-genetic factors like exposure to occupational vibration [18,19] have been investigated in different populations with inconsistent results. Discrepancies in the reported findings [20] could derive from the lack of a consistent definition of the lumbar spine pathological phenotypes analyzed and by the differences associated with the specific ethnic group examined.

Very recently, in an Italian case-control study, we reported that the *VDR*-FokI polymorphism and conventional, behavioral and environmental factors were associated with lumbar spine pathologies [21]. In the previous work we evaluated the overall population of cases in comparison with asymptomatic controls, without taking into account possible gender effects. However, sex-related differences were noted recently in several diseases [22] and in particular in musculoskeletal disorders [23]. Interestingly, some gender differences were noted when reporting spine pain, with females being more likely to report neck and back pain, and males being more likely to have a higher number of days absent from work and diagnosed hernia [24]. Thus, sex-unstratified analyses could lead to missing specific gender effects in the observed association of spine disorders and risk factors.

To our knowledge, no study explored the gender-related interplay between the *VDR*-FokI polymorphism and conventional risk factors predisposing to lumbar spine pathologies in the Italian population. The aims of this study were to evaluate in *VDR*-FokI frequency distributions and conventional risk factors separately in Italian males and females with specific lumbar spine pathologies compared to asymptomatic controls.

## 2. Results

### 2.1. Influences of Conventional, Behavioral and Environmental Risk Factors According to Gender

The putative non-genetic categorical risk factors associated to all lumbar spine diseases in the overall population and in males or females were shown in Table 1. Cases and controls did not differ by gender (*p* = 0.19). Males and females did not differ by age (*p* = 0.70).

In the overall cohort of cases, age over 45 years or over 50 years, overweight or obesity, family history, past and present smoking habit, present smoking ≥10 cigarettes per day, and more than sedentary or intense job physical demand were all significantly associated with the development of lumbar spine pathologies, with significant ORs (odds ratio) ranging approximately from 2 to 3. Of note, present smoking ≥20 cigarettes per day had an elevated OR = 6.05; 95% CI (95% confidence interval) = 1.76–20.8; *p* = 0.001. A strong dose-response effect was observed concerning the increasing numbers of hours/day of exposure to vehicular vibration; the increased risk to develop spine pathologies ranged from a 1.6-fold at >1 h/day, to 2.9-fold at >2 h/day, to 3.7-fold at >3 h/day and to 8.5-fold increased risk at >4 h/day of vibration exposure. In our Italian cohort, controls subjects practiced leisure physical activity at least once per week 2-fold more frequently than the pathological subjects (OR = 1.98; 95% CI = 1.50–2.62; *p* < 0.001).

Almost all the associations found for the entire cohort were confirmed for male patients compared to male controls, the only exceptions were an age ≥50 years and present smoking (both these categories corresponded to tendencies in males). Notably, family history was a six-fold risk factor for males. Unexpectedly, for female patients compared to female controls the only conventional risk factors significantly associated with an approximately 2- to 3-fold increased risk for lumbar spine pathologies were age ≥45 to ≥50 years, overweight, and family history. Additionally, in females, a lower frequency of spine pathologies was associated with leisure physical activity once or more or twice or more per week. Thus, a remarkable gender difference was noted in regard to association of lumbar spine disease with physical job demand and exposure to vibrations. Particularly, intense physical job demand was associated to a six-fold increased risk for men, while it was indifferent for women. However, it is to note that, in our cohort, males and females differed in frequency of intense physical job demand (*p* = 0.003). Women had in general less risky behaviors, for example, percentages of smokers were not different between genders, but males more than females smoked ≥10 cigarettes/day (*p* = 0.023) or ≥20 cigarettes/day (*p* = 0.030), indeed, only very few females (*n* = 5) smoked ≥20 cigarettes/day *vs.* 16 males. Moreover, women were exposed to less severe environmental risk factors, exposure to vibration over 1 h/day was more frequent in males than in females (*p* < 0.001), notably only 1 woman (a case) in the entire sample was exposed to more than 4 h of vibrations/day *vs.* 35 males (31 cases and 4 controls), *p* < 0.001.

### 2.2. VDR-FokI Genotypes and Alleles According to Gender

Data concerning the FokI genotypes in the entire cohort of 521 subjects (267 cases and 254 controls) and gender-related were reported in Table 2. Male and female cases and controls did not differ in genotype frequencies. The *VDR*-FokI genotypes were in Hardy-Weinberg equilibrium (HWE) in male controls (χ^2^ = 0.49, *p* = 0.48), female controls (χ^2^ = 0.15, *p* = 0.69), male cases (χ^2^ = 0.38, *p* = 0.54), and female cases (χ^2^ = 0.11, *p* = 0.74).

Concerning the genotype frequencies of all pathological cases compared with controls, we did not observe significant differences, with the exception of a just significant lower frequency of the homozygous *ff* genotype in male cases (8.1%) than in male controls (15.7%); crude OR = 0.47; 95% CI = 0.22–1.00; *p* = 0.047). However, the observed significant difference for *ff* in males was lost after adjustment for conventional risk factors. Moreover, in the male cohort there was a tendency for an association of the *F* allele with lumbar spine pathologies (69.8% of cases *vs.* 62.2% of controls; crude OR = 1.40; 95% CI = 0.99–2.00; *p* = 0.060); thus, for the *f* allele there was a protective tendency.

Confirmatory with our previous investigation [21], by cumulative analysis including both genders, significant *FF* associations were found for some specific patients; Subgroup 2 (*i.e.*, discopathies and/or osteochondrosis with disc herniation), crude OR = 1.95; 95% CI = 1.19–3.20, *p* = 0.007; adjusted OR = 2.09; 95% CI = 1.19–3.66, *p* = 0.011, and Subgroup C (*i.e.*, all discopathies), crude OR = 2.08; 95% CI = 1.19–3.62, *p* = 0.009; adjusted OR = 1.88; 95% CI = 1.03–3.43, *p* = 0.039. The *ff* genotype was protective in crude analysis only for Subgroup B (*i.e.*, all discopathies and/or osteochondrosis), crude OR = 0.46; 95% CI = 0.22–0.99, *p* = 0.043, adjusted OR = 0.42; 95% CI = 0.18–1.00, *p* = 0.051; and for Subgroup D (*i.e.*, all osteochondrosis) crude OR = 0.25; 95% CI = 0.06–1.08, *p* = 0.047, adjusted OR = 0.23; 95% CI = 0.05–1.17, *p* = 0.078. The complete data analysis of genotype association in different pathologic Subgroups not stratified for sex was reported in Supplementary Table S1.

Gender-related analysis of genotypes in pathological Subgroups was shown in Table 3 (males) and Table 4 (females). Regarding the male population (Table 3), in Subgroup 2 the *FF* genotype had a frequency of 52.7% *vs.* 40.2% of male controls (crude OR not significant; adjusted OR = 2.32; 95% CI = 1.02–5.30, *p* = 0.045). Moreover, in Subgroup A and C the *FF* genotype had a tendency for risk. In males, the *ff* genotype was protective in crude analysis for Subgroup A (crude OR = 0.39; 95% CI = 0.16–0.96, *p* = 0.036) and Subgroup 1 + 2 + 3 (all patients excluding stenosis and/or spondilolysthesis) (crude OR = 0.42; 95% CI = 0.18–0.96, *p* = 0.035). Finally, the *ff* genotype was protective for Subgroup B in adjusted analysis (adjusted OR = 0.23; 95% CI = 0.06–0.88, *p* = 0.032).

**Table 1:** Categorical risk factors for all subjects (*n* = 521), and for males (*n* = 276) or females (*n* = 245) controls and cases.

Categorical Variable	All Controls	All Cases	OR (95% CI)	Male Controls	Male Cases	OR (95% CI)	Female Controls	Female Cases	OR (95% CI)
	*n* = 254	*n* = 267	*p* Value	*n* = 127 (50.0%)	*n* = 149 (55.8%)	*p* Value	*n* = 127 (50.0%)	*n* = 118 (44.2%)	*p* Value
Age ≥ 45 years	79 (31.1)	136 (50.9)	2.30 (1.61–3.29) *p* < 0.001	41 (32.3)	72 (48.3)	1.96 (1.20–3.21) *p* = 0.007	38 (29.9)	64 (54.2)	2.78 (1.64–4.69) *p* < 0.001
Age ≥ 50 years	45 (17.7)	83 (31.1)	2.10 (1.39–3.17) *p* < 0.001	23 (18.1)	40 (26.8)	1.66 (0.93–2.96) *p* = 0.085	22 (17.3)	43 (36.4)	2.74 (1.51–4.95) *p* = 0.001
BMI ≥ 25.0 kg/m^2^	87 (34.3)	132 (49.4)	1.88 (1.32–2.67) *p* < 0.001	61 (48.0)	92 (61.7)	1.75 (1.08–2.82) *p* = 0.022	26 (20.5)	40 (33.9)	1.99 (1.12–3.54) *p* = 0.018
BMI ≥ 30.0 kg/m^2^	17 (6.7)	37 (13.9)	2.24 (1.23–4.10) *p* = 0.007	11 (8.7)	27 (18.1)	2.33 (1.11–4.92) *p* = 0.023	6 (4.7)	10 (8.5)	1.87 (0.66–5.31)
Family history	37 (14.6)	97 (36.3)	3.35 (2.18–5.14) *p* < 0.001	13 (10.2)	61 (40.9)	6.08 (3.14–11.8) *p* < 0.001	24 (18.9)	36 (30.5)	1.88 (1.04–3.41) *p* = 0.035
Past and present smoking	104 (40.9)	144 (53.9)	1.69 (1.19–2.39) *p* = 0.003	61 (48.0)	93 (62.4)	1.80 (1.11–2.91) *p* = 0.016	43 (33.9)	51 (43.2)	1.49 (0.89–2.50)
Present smoking	57 (22.4)	86 (32.2)	1.64 (1.11–2.43) *p* = 0.013	31 (24.4)	52 (34.9)	1.66 (0.98–2.81) *p* = 0.058	26 (20.5)	34 (28.8)	1.57 (0.87–2.83)
Smoking ≥ 10 cigarettes/day	23 (9.1)	54 (20.2)	2.55 (1.51–4.29) *p* < 0.001	12 (9.4)	38 (25.5)	3.28 (1.63–6.60) *p* = 0.001	11 (8.7)	16 (13.6)	1.65 (0.73–3.73)
Smoking ≥ 20 cigarettes/day	3 (1.2)	18 (6.7)	6.05 (1.76–20.8) *p* = 0.001	1 (0.8)	15 (10.1)	14.1 (1.84–108) *p* = 0.001	2 (1.6)	3 (2.5)	1.63 (0.27–9.93)
Physical job demand more than sedentary	155 (61.0)	192 ^a^ (73.3)	1.75 (1.21–2.54) *p* = 0.003	71 (55.9)	110 (74.8) ^b^	2.35 (1.41–3.91) *p* = 0.001	84 (66.1)	82 (71.3) ^d^	1.27 (0.74–2.20)
Medium or intense	91 (35.8)	117 ^a^ (44.7)	1.45 (1.02–2.06) *p* = 0.041	40 (31.5)	80 (54.4) ^b^	2.60 (1.58–4.26) *p* < 0.001	51 (40.2)	37 (32.2) ^d^	0.71 (0.42–1.20)
Intense	21 (8.3)	53 ^a^ (20.2)	2.81 (1.64–4.82) *p* < 0.001	8 (6.3)	43 (29.3) ^b^	6.15 (2.77–13.7) *p* < 0.001	13 (10.2)	10 (8.7) ^d^	0.84 (0.35–1.99)
Exposure to vibration > 0 h/day	219 (86.2)	218 (81.6)	0.71 (0.44–1.14)	111 (87.4)	127 (85.2)	0.83 (0.42–1.66)	108 (85.0)	91 (77.1)	0.59 (0.31–1.14)
Exposure to vibration > 1 h/day	89 (35.0)	124 (46.4)	1.61 (1.13–2.29) *p* = 0.008	47 (37.0)	86 (57.7)	2.32 (1.43–3.77) *p* = 0.001	42 (33.1)	38 (32.2)	0.96 (0.56–1.64)
Exposure to vibration > 2 h/day	26 (10.2)	66 (20.7)	2.88 (1.76–4.71) *p* < 0.001	14 (11.0)	52 (34.9)	4.33 (2.26–8.28) *p* < 0.001	12 (9.4)	14 (11.9)	1.29 (0.57–2.92)
Exposure to vibration > 3 h/day	13 (5.1)	44 (16.5)	3.66 (1.92–6.97) *p* < 0.001	7 (5.5)	39 (26.2)	6.08 (2.61–14.2) *p* < 0.001	6 (4.7)	5 (4.2)	0.89 (0.27–3.01)
Exposure to vibration > 4 h/day	4 (1.6)	32 (12.0)	8.51 (2.97–24.4) *p* < 0.001	4 (3.1)	31 (20.8)	8.08 (2.77–23.6) *p* < 0.001	0 (0)	1 (0.8)	-
Leisure physical activity once or more per week	148 (58.3)	85 (31.8)	0.34 (0.24–0.48) *p* < 0.001	81 (63.8)	59 (39.9) ^c^	0.38 (0.23–0.61) *p* < 0.001	67 (52.8)	26 (22.0)	0.25 (0.15–0.44) *p* < 0.001
Leisure physical activity 2-fold or more per week	104 (40.9)	55 (20.6)	0.38 (0.26–0.55) *p* < 0.001	60 (47.2)	40 (27.0) ^c^	0.41 (0.25–0.68) *p* = 0.001	44 (34.6)	15 (12.7)	0.28 (0.14–0.53) *p* < 0.001

OR: odds ratio; 95% CI: 95% confidence interval; BMI: body mass index; ^a^ 5 patients had missing information about intensity of physical demand at work, thus a total of 262 patients’ data were available; ^b^ 2 male patients had missing information about intensity of physical demand at work, thus a total of 147 data were available; ^c^ 1 male patient had missing information about leisure physical activity per week, thus a total of 148 data were available; ^d^ 3 female patients had missing information about intensity of physical demand at work, thus a total of 115 data were available; Only significant *p* ≤ 0.05 or tendency *p* ≤ 0.10 were indicated.

**Table 2:** Association of *VDR*-FokI genotypes and alleles in healthy controls and cases, and in males and females.

Variables		Controls *n* = 254 (%)			Cases *n* = 267 (%)			Crude OR (95% CI) *p* Value			Adjusted OR ^1^ (95% CI) *p* Value		
		All Subjects *n* = 254	Males *n* = 127 (50.0)	Females *n* = 127 (50.0)	All Subjects *n* = 267	Males *n* = 149 (55.8)	Females *n* = 118 (44.2)	All Subjects *n* = 521	Males *n* = 276	Females *n* = 245	All Subjects	Males	Females
*VDR*-FokI genotypes	*FF*	101 (39.8)	51 (40.2)	50 (39.4)	117 (43.8)	71 (47.7)	46 (39.0)	1.18 (0.83–1.68)	1.36 (0.84–2.19)	0.98 (0.59–1.64)	1.13 (0.77–1.66)	1.40 (0.79–2.48)	1.08 (0.62–1.88)
	*Ff*	117 (46.1)	56 (44.1)	61 (48.0)	120 (44.9)	66 (44.3)	54 (45.8)	0.96 (0.68–1.35)	1.01 (0.63–1.62)	0.91 (0.55–1.51)	0.94 (0.64–1.38)	0.97 (0.55–1.72)	0.81 (0.47–1.39)
	*ff*	36 (14.2)	20 (15.7)	16 (12.6)	30 (11.2)	12 (8.1)	18 (15.3)	0.77 (0.46–1.29)	0.47 (0.22–1.00) *p* = 0.047	1.25 (0.60–2.58)	0.89 (0.50–1.58)	0.48 (0.19–1.20)	1.34 (0.61–2.92)
*VDR*-FokI alleles	*F*	319/508 (62.8)	158/254 (62.2)	161/254 (63.4)	354/534 (66.3)	208/298 (69.8)	146/236 (61.9)	1.17 (0.90–1.50)	1.40 (0.99–2.00) *p* = 0.060	0.94 (0.65–1.35)	1.10 (0.83–1.45)	1.42 (0.93–2.17)	0.97 (0.65–1.43)
	*f*	189/508 (37.2)	96/254 (37.8)	93/254 (36.6)	180/534 (33.7)	90/298 (30.2)	90/236 (38.1)	0.86 (0.67–1.11)	0.71 (0.50–1.01) *p* = 0.060	1.07 (0.74–1.54)	0.91 (0.69–1.21)	0.71 (0.46–1.08)	1.04 (0.70–1.53)

^1^ Adjusted OR: multivariate analysis; OR adjusted for age; BMI, family history, smoking, physical job demand and exposure to vibrations; Only significant *p* ≤ 0.05 or tendency *p* ≤ 0.10 were indicated.

**Table 3:** Association of *VD*R FokI genotypes with lumbar spine pathologies in males. Total 276 male subjects (127 controls and 149 cases) evaluated. Male patients were subdivided in Subgroups 1 to 4 and A to D according to specific pathologic conditions Subgroup 1 = patients with disc herniation alone; Subgroup 2 = patients with discopathies and/or osteochondrosis associated with disc herniation; Subgroup 3 = patients with discopathies and/or osteochondrosis without herniation; and Subgroup 4 = patients with stenosis and/or spondilolysthesis. Subgroup A, Subgroup 1 grouped with Subgroup 2 (*i.e.*, all hernia cases with or without concomitant additional conditions); Subgroup B, Subgroup 2 grouped with Subgroup 3 (*i.e.*, all discopathies and/or osteochondrosis); Subgroup C, all discopathies cases with or without concomitant disc herniation; Subgroup D, all osteochondrosis cases with or without concomitant disc herniation.

Variables	*FF* *n* (%)	Crude OR (95% CI) *p* Value	Adjusted OR ^1^ (95% CI) *p* Value	*Ff* *n* (%)	Crude OR (95% CI) *p* Value	Adjusted OR ^1^ (95% CI) *p* Value	*ff* *n* (%)	Crude OR (95% CI) *p* Value	Adjusted OR ^1^ (95% CI) *p* Value
Controls *n* =127	51 (40.2)	-	-	56 (44.1)	-	-	20 (15.7)	-	-
Subgroup 1 *n* = 48	25 (52.1)	1.62 (0.83–3.16)	1.46 (0.65–3.27)	19 (39.6)	0.83 (0.42–1.63)	0.82 (0.37–1.84)	4 (8.3)	0.49 (0.16–1.51)	0.62 (0.18–2.22)
Subgroup 2 *n* = 55	29 (52.7)	1.66 (0.88–3.14)	2.32 (1.02–5.30) *p* = 0.045	23 (41.8)	0.91 (0.48–1.73)	0.72 (0.32–1.62)	3 (5.5)	0.31 (0.09–1.09) *p* = 0.055	0.24 (0.05–1.14) *p* = 0.073
Subgroup 3 *n* = 21	7 (33.3)	0.75 (0.28–1.97)	0.70 (0.22–2.23)	12 (57.1)	1.69 (0.67–4.30)	2.34 (0.75–7.31)	2 (9.5)	0.56 (0.12–2.61)	0.30 (0.04–2.04)
Subgroup 4 *n* = 25	10 (40.0)	0.99 (0.41–2.38)	0.93 (0.33–2.58)	12 (48.0)	1.17 (0.50–2.76)	1.24 (0.47–3.31)	3 (12.0)	0.73 (0.20–2.67)	0.80 (0.19–3.37)
Subgroup 1 + 2 + 3 *n* = 124	61 (49.2)	1.44 (0.88–2.38)	1.48 (0.81–2.69)	54 (43.5)	0.98 (0.59–1.61)	0.95 (0.52–1.74)	9 (7.3)	0.42 (0.18–0.96) *p* = 0.035	0.43 (0.16–1.17) *p* = 0.097
Subgroup A *n* = 103	54 (52.4)	1.64 (0.97–2.78) *p* = 0.063	1.80 (0.94–3.43) *p* = 0.074	42 (40.8)	0.87 (0.52–1.48)	0.75 (0.39–1.44)	7 (6.8)	0.39 (0.16–0.96) *p* = 0.036	0.47 (0.16–1.36)
Subgroup B *n* = 76	36 (47.4)	1.34 (0.76–2.38)	1.59 (0.78–3.22)	35 (46.1)	1.08 (061–1.92)	1.06 (0.53–2.14)	5 (6.6)	0.38 (0.14–1.05) *p* = 0.054	0.23 (0.06–0.88) *p* = 0.032
Subgroup C *n* = 27	16 (59.3)	2.17 (0.93–5.05) *p* = 0.069	2.59 (0.89–7.50) *p* = 0.079	10 (37.0)	0.75 (0.32–1.76)	0.67 (0.23–1.90)	1 (3.7)	0.21 (0.03–1.60) *p* = 0.098	0.18 (0.02–1.84)
Subgroup D *n* = 40	19 (47.5)	1.35 (0.66–2.76)	1.94 (0.79–4.79)	19 (47.5)	1.15 (0.56–2.34)	0.90 (0.37–2.16)	2 (5.0)	0.28 (0.06–1.26) *p* = 0.080	0.22 (0.04–1.31) *p* = 0.096

^1^ Adjusted OR: multivariate analysis; OR adjusted for age; BMI, family history, smoking, physical job demand and exposure to vibrations; Only significant *p* ≤ 0.05 or tendency *p* ≤ 0.10 were indicated.

**Table 4:** Association of *VDR* FokI genotypes and lumbar spine pathologies in females. Total 245 female subjects (127 controls and 118 cases) evaluated. Female patients were subdivided in Subgroups 1 to 4 and A to D according to specific pathologic conditions. Subgroup 1 = patients with disc herniation alone; Subgroup 2 = patients with discopathies and/or osteochondrosis associated with disc herniation; Subgroup 3 = patients with discopathies and/or osteochondrosis without herniation; and Subgroup 4 = patients with stenosis and/or spondilolysthesis. Subgroup A, Subgroup 1 grouped with Subgroup 2 (*i.e.*, all hernia cases with or without concomitant additional conditions); Subgroup B, Subgroup 2 grouped with Subgroup 3; Subgroup C, all discopathies cases with or without concomitant disc herniation; Subgroup D, all osteochondrosis cases with or without concomitant disc herniation.

Variables	*FF* *n* (%)	Crude OR	Adjusted OR ^1^	*Ff* *n* (%)	Crude OR	Adjusted OR ^1^	*ff* *n* (%)	Crude OR	Adjusted OR ^1^
		(95% CI)	(95% CI)		(95% CI)	(95% CI)		(95% CI)	(95% CI)
		*p* Value	*p* Value		*p* Value	*p* Value		*p* Value	*p* Value
Controls *n* = 127	50 (39.4)	-	-	61 (48.0)	-	-	16 (12.6)	-	-
Subgroup 1 *n* = 41	12 (29.3)	0.64 (0.30–1.36)	0.75 (0.34–1.66)	21 (51.2)	1.14 (0.56–2.30)	0.96 (0.45–2.04)	8 (19.5)	1.68 (0.66–4.28)	1.81 (0.65–4.99)
Subgroup 2 *n* = 32	20 (62.5)	2.57 (1.15–5.71) *p* = 0.018	2.48 (1.07–5.74) *p* = 0.034	9 (28.1)	0.42 (0.18–0.99) *p* = 0.043	0.38 (0.16–0.93) *p* = 0.033	3 (9.4)	0.72 (0.20–2.63)	1.02 (0.25–4.06)
Subgroup 3 *n* = 19	7 (36.8)	0.90 (0.33–2.44)	0.90 (0.32–2.54)	11 (57.9)	1.49 (0.56–3.94)	1.41 (0.50–3.96)	1 (5.3)	0.39 (0.05–3.09)	0.43 (0.05–3.72)
Subgroup 4 *n* = 26	7 (26.9)	0.57 (0.22–1.45)	0.55 (0.19–1.57)	13 (50.0)	1.08 (0.47–2.52)	0.89 (0.34–2.30)	6 (23.1)	2.08 (0.73–5.96)	3.20 (0.94–11.0) *p* = 0.064
Subgroup 1 + 2 + 3 *n* = 92	39 (42.4)	1.13 (0.66–1.96)	1.25 (0.70–2.24)	41 (44.6)	0.87 (0.51–1.49)	0.77 (0.43–1.37)	12 (13.0)	1.04 (0.47–2.32)	1.10 (0.46–2.59)
Subgroup A *n* = 73	32 (43.8)	1.20 (0.67–2.15)	1.30 (0.69–2.42)	30 (41.1)	0.76 (0.42–1.35)	0.67 (0.36–1.25)	11 (15.1)	1.23 (0.54–2.82)	1.39 (0.56–3.44)
Subgroup B *n* = 51	27 (52.9)	1.73 (0.90–3.34) *p* = 0.098	1.74 (0.88–3.46)	20 (39.2)	0.70 (0.36–1.35)	0.64 (0.32–1.28)	4 (7.8)	0.59 (0.19–1.86)	0.71 (0.21–2.36)
Subgroup C *n* = 37	21 (56.8)	2.02 (0.96–4.24) *p* = 0.060	1.88 (0.87–4.08)	12 (32.4)	0.52 (0.24–1.12) *p* = 0.093	0.49 (0.22–1.10) *p* = 0.083	4 (10.8)	0.84 (0.26–2.69)	1.07 (0.31–3.65)
Subgroup D *n* = 10	5 (50.0)	1.54 (0.42–5.59)	1.62 (0.43–6.06)	5 (50.0)	1.08 (0.30–3.92)	1.01 (0.26–3.85)	0 (-)	-	-

^1^ Adjusted OR: multivariate analysis; OR adjusted for age; BMI, family history, smoking, physical job demand and exposure to vibrations; Only significant *p* ≤ 0.05 or tendency *p* ≤ 0.10 were indicated.

Regarding the female population (Table 4) in Subgroup 2 the *FF* genotype had a frequency of 62.5% *vs.* 39.4% of female controls (crude OR = 2.57; 95% CI = 1.15–5.71, *p* = 0.018; adjusted OR = 2.48, 95% CI = 1.07–5.74, *p* = 0.034), while the heterozygous *Ff* genotype was protective (crude OR = 0.42; 95% CI = 0.18–0.99, *p* = 0.043; adjusted OR = 0.38, 95% CI = 0.16–0.93, *p* = 0.033). No other significant finding was observed for FokI genotypes in female Subgroups. An intriguing observation was that the *ff* genotype showed a tendency for risk in the female Subgroup 4 (adjusted OR = 3.20, 95% CI = 0.94–11.0, *p* = 0.064).

FokI alleles associations with detailed lumbar pathologic conditions in the entire sample including both sexes are reported in the Supplementary file (Supplementary Table S2). The *F* allele was a risk factor for several Subgroups of patients (and consequently the *f* allele was protective) as follow: Subgroup 2 (crude OR = 1.75; 95% CI = 1.19–2.58, *p* = 0.004; adjusted OR = 1.76; 95% CI = 1.14–2.73, *p* = 0.011); Subgroup A (crude OR = 1.34; 95% CI = 1.00–1.79, *p* = 0.048; adjusted OR not significant); Subgroup B (crude OR = 1.47; 95% CI = 1.06–2.04, *p* = 0.020; adjusted OR = 1.49; 95% CI = 1.04–2.13, *p* = 0.032), and Subgroup C (crude OR = 1.78; 95% CI = 1.15–2.76, *p* = 0.010; adjusted OR = 1.64; 95% CI = 1.02–2.62, *p* = 0.040).

Gender related FokI alleles associations with detailed lumbar pathologic conditions were reported in Table 5 (males) and Table 6 (females).

In males (Table 5) frequency of the *F* allele was almost 2-fold higher in several Subgroups of patients. Specifically, Subgroup 2 (crude OR = 1.70; 95% CI = 1.04–2.78, *p* = 0.035; adjusted OR = 2.16; 95% CI = 1.15–4.05, *p* = 0.017), Subgroup 1 + 2 + 3 (crude OR = 1.49; 95% CI = 1.02–2.16, *p* = 0.038; adjusted OR = 1.50; 95% CI = 0.95–2.35, *p* = 0.080), Subgroup A (crude OR = 1.63; 95% CI = 1.09–2.42, *p* = 0.016; adjusted OR = 1.66; 95% CI = 1.02–2.70, *p* = 0.042), Subgroup B (crude OR = 1.45; 95% CI = 0.94–2.22, *p* = 0.093; adjusted OR = 1.72; 95% CI = 1.01–2.95, *p* = 0.048), and Subgroup C (crude OR = 2.13; 95% CI = 1.07–4.24, *p* = 0.029; adjusted OR = 2.36; 95% CI = 1.02–5.44, *p* = 0.044) in respect to male controls. Consequently the *f* allele was protective for male patients of Subgroups 2, 1 + 2 + 3, A, B, and C.

As shown in Table 6, no significant finding was observed for FokI alleles in specific subgroups of female patients, with the exception of Subgroup 2 for which the *F* allele (frequency 76.6% in female cases *vs.* 63.4% in female controls) in a crude analysis had almost 2-fold increased risk (crude OR = 1.89; 95% CI = 1.00–3.55, *p* = 0.047; adjusted OR not significant). Consequently the *f* allele in females was protective for Subgroup 2, although significance was lost in adjusted analysis. Of note, an inverse trend was observed for the *f* allele in female Subgroup 4 (*f* frequency 48.1% *vs.* 36.6% of female controls), crude OR not significant; adjusted OR = 1.82; 95% CI = 0.92–3.59; *p* = 0.085. Overall in our study, the Subgroup 4 (*i.e.*, stenosis and/or spondilolysthesis) seems to differ in respect to all other Subgroups.

**Table 5:** Association of *VDR* FokI alleles with lumbar spine pathologies in males. Total 276 male subjects (127 controls and 149 cases) evaluated. Male patients were subdivided in Subgroups 1 to 4 and A to D according to specific pathologic conditions. Subgroup 1 = patients with disc herniation alone; Subgroup 2 = patients with discopathies and/or osteochondrosis associated with disc herniation; Subgroup 3 = patients with discopathies and/or osteochondrosis without herniation; and Subgroup 4 = patients with stenosis and/or spondilolysthesis. Subgroup A, Subgroup 1 grouped with Subgroup 2 (*i.e.*, all hernia cases with or without concomitant additional conditions); Subgroup B, Subgroup 2 grouped with Subgroup 3; Subgroup C, all discopathies cases with or without concomitant disc herniation; Subgroup D, all osteochondrosis cases with or without concomitant disc herniation.

Male Subjects						
Variables	*F* *n* (%)	Crude OR (95% CI) *p* Value	Adjusted OR ^1^ (95% CI) *p* Value	*f* *n* (%)	Crude OR (95% CI) *p* Value	Adjusted OR ^1^ (95% CI) *p* Value
Controls *n* = 254	158 (62.2)	-	-	96 (37.8)	-	-
Subgroup 1 *n* = 96	69 (71.9)	1.55 (0.93–2.59) *p* = 0.091	1.37 (0.75–2.50)	27 (28.1)	0.64 (0.39–1.08) *p* = 0.091	0.73 (0.40–1.34)
Subgroup 2 *n* = 110	81 (73.6)	1.70 (1.04–2.78) *p* = 0.035	2.16 (1.15–4.05) *p* = 0.017	29 (26.4)	0.59 (0.36–0.97) *p* = 0.035	0.46 (0.25–0.87) *p* = 0.017
Subgroup 3 *n* = 42	26 (61.9)	0.99 (0.50–1.93)	1.09 (0.50–2.37)	16 (38.1)	1.01 (0.52–1.98)	0.92 (0.42–2.01)
Subgroup 4 *n* = 50	32 (64.0)	1.08 (0.58–2.03)	1.01 (0.49–2.09)	18 (36.0)	0.93 (0.49–1.74)	0.99 (0.48–2.03)
Subgroup 1 + 2 + 3 *n* = 248	176 (71.0)	1.49 (1.02–2.16) *p* = 0.038	1.50 (0.95–2.35) *p* = 0.080	72 (29.0)	0.67 (0.46–0.98) *p* = 0.038	0.67 (0.43–1.05) *p* = 0.080
Subgroup A *n* = 206	150 (72.8)	1.63 (1.09–2.42) *p* = 0.016	1.66 (1.02–2.70) *p* = 0.042	56 (27.2)	0.61 (0.41–0.92) *p* = 0.016	0.60 (0.37–0.98) *p* = 0.042
Subgroup B *n* = 152	107 (70.4)	1.45 (0.94–2.22) *p* = 0.093	1.72 (1.01–2.95) *p* = 0.048	45 (29.6)	0.69 (0.45–1.07) *p* = 0.093	0.58 (0.34–0.99) *p* = 0.048
Subgroup C *n* = 54	42 (77.8)	2.13 (1.07–4.24) *p* = 0.029	2.36 (1.02–5.44) *p* = 0.044	12 (22.2)	0.47 (0.24–0.94) *p* = 0.029	0.42 (0.18–0.98) *p* = 0.044
Subgroup D *n* = 80	57 (71.3)	1.51 (0.87–2.60)	1.92 (0.97–3.80) *p* = 0.060	23 (28.8)	0.66 (0.38–1.15)	0.52 (0.26–1.03) *p* = 0.060

^1^ Adjusted OR: multivariate analysis; OR adjusted for age; BMI, family history, smoking, physical job demand and exposure to vibrations; Only significant *p* ≤ 0.05 or tendency *p* ≤ 0.10 were indicated.

**Table 6:** Association of *VDR* FokI alleles with lumbar spine pathologies in females. Total 245 female subjects (127 controls and 118 cases) evaluated. Female patients were subdivided in Subgroups 1 to 4 and A to D according to specific pathologic conditions. Subgroup 1 = patients with disc herniation alone; Subgroup 2 = patients with discopathies and/or osteochondrosis associated with disc herniation; Subgroup 3 = patients with discopathies and/or osteochondrosis without herniation; and Subgroup 4 = patients with stenosis and/or spondilolysthesis. Subgroup A, Subgroup 1 grouped with Subgroup 2 (*i.e*., all hernia cases with or without concomitant additional conditions); Subgroup B, Subgroup 2 grouped with Subgroup 3; Subgroup C, all discopathies cases with or without concomitant disc herniation; Subgroup D, all osteochondrosis cases with or without concomitant disc herniation.

Female Subjects						
Variables	*F* *n* (%)	Crude OR (95% CI) *p* Value	Adjusted OR ^1^ (95% CI) *p* Value	*f* *n* (%)	Crude OR (95% CI) *p* Value	Adjusted OR ^1^ (95% CI) *p* Value
Controls *n* = 254	161 (63.4)	-	-	93 (36.6)	-	-
Subgroup 1 *n* = 82	45 (54.9)	0.70 (0.42–1.16)	0.75 (0.44–1.28)	37 (45.1)	1.42 (0.86–2.36)	1.33 (0.78–2.27)
Subgroup 2 *n* = 64	49 (76.6)	1.89 (1.00–3.55) *p* = 0.047	1.71 (0.89–3.28)	15 (23.4)	0.53 (0.28–1.00) *p* = 0.047	0.59 (0.31–1.13)
Subgroup 3 *n* = 38	25 (65.8)	1.11 (0.54–2.28)	1.08 (0.52–2.26)	13 (34.2)	0.90 (0.44–1.84)	0.93 (0.44–1.94)
Subgroup 4 *n* = 52	27 (51.9)	0.62 (0.34–1.14)	0.55 (0.28–1.09) *p* = 0.085	25 (48.1)	1.60 (0.88–2.92)	1.82 (0.92–3.59) *p* = 0.085
Subgroup 1 + 2+ 3 *n* = 184	119 (64.7)	1.06 (0.71–1.57)	1.10 (0.73–1.67)	65 (35.3)	0.95 (0.64–1.41)	0.91 (0.60–1.38)
Subgroup A *n* = 146	94 (64.4)	1.04 (0.68–1.60)	1.06 (0.68–1.67)	52 (35.6)	0.96 (0.63–1.46)	0.94 (0.60–1.48)
Subgroup B *n* = 102	74 (72.5)	1.53 (0.92–2.53) *p* = 0.099	1.45 (0.86–2.44)	28 (27.5)	0.66 (0.40–1.09) *p* = 0.099	0.69 (0.41–1.16)
Subgroup C *n* = 74	54 (73.0)	1.56 (0.88–2.77)	1.40 (0.77–2.53)	20 (27.0)	0.64 (0.36–1.14)	0.72 (0.40–1.29)
Subgroup D *n* = 20	15 (75.0)	1.73 (0.61–4.92)	1.74 (0.60–5.00)	5 (25.0)	0.58 (0.20–1.64)	0.58 (0.20–1.66)

^1^ Adjusted OR: multivariate analysis; OR adjusted for age; BMI, family history, smoking, physical job demand and exposure to vibrations; Only significant *p* ≤ 0.05 or tendency *p* ≤ 0.10 were indicated.

## 3. Discussion

Accumulating evidence highlights that gender-related medicine may have a major impact in evaluation of incidence, prognosis and optimal treatment of several diseases. Notably, gender disparities were evident in risk factors for several pathologic conditions including osteoporosis, cancer and cardiovascular diseases [22]. However, gender differences in lumbar spine pathologies were minimally explored [23]; no study, to our knowledge, investigated this issue in the Italian population.

In our present study, interesting results emerged from the analysis of the association of non-genetic conventional, behavioral and environmental categorical variables with development of lumbar spine pathologies in the overall cohort of cases and in both sexes compared to appropriate controls. In the entire sample, we observed an association between lumbar spine pathologies and higher number of hours/day exposure to vibration with a strong dose-response effect, a more physically demanding rather than sedentary job, and the physical intensity of a job. Notably, this risk profile was confirmed only in males. Among the other putative conventional risk factors analyzed, we observed that, in our entire sex-unstratified pathological sample, family history, higher age (≥45 years), overweight (≥25.0 kg/m^2^) and smoking habits were associated with an approximately 2-fold risk for lumbar spine pathologies. Of note, heavy smoking (≥20 cigarettes/day) was associated with a six-fold increased risk. These findings concerning smoking habits are in accordance with previous investigations showing that smoking can accelerate disc degeneration and induce back pain [25,26], and is also an important risk factor for recurrent disc herniation [27].

Overall, our findings highlighted that voluntary behaviors, in addition to environmental factors, are major determinants in lumbar spine pathologies. Notably, we found that women appeared to have less of the noted risky behaviors.

Interestingly, all the conventional risk factors observed in the sex-unstratified cohort were associated with a pathological phenotype in male patients, while in females patients only family history, higher age, overweight and lower voluntary practice of leisure physical activity were determinant of risk. In general, the female population was less exposed to environmental and occupational risk factors, which could account for a gender-specific influence regarding the pathological phenotype. In line with our findings, a different prevalence for risk factors associated to back pain according to gender was observed by other authors in the Canadian (Quebec) population [23].

In a recently published paper we determined FokI genotypes and alleles in this sample of lumbar spine pathologic Italian white subjects enrolled in Milan (Northern Italy) [21]. We now compared them with 254 healthy controls including equal number of males and females.

Analysis of FokI polymorphism considering the heterogeneous group comprising all patients having lumbar pathologies according to gender did not reveal significant findings with the exception of a just significant protective effect of *ff* genotype and a tendency for protection of the *f* allele in males. Increasing evidence suggests that specific subgroups of patients with specific pathological features should be investigated separately, thus, we adopted the same approach used in our previous paper [21]. 

Confirmatory with previous findings, *FF* genotype was associated with roughly a two-fold increased risk for discopathies and/or osteochondrosis concomitant with herniation after adjusting for risk factors in the entire population, and also in separately analyzed males and females. By adjusted analysis, the *FF* genotype was associated with roughly a two-fold increased risk for discopathies in general in the entire population, but considering the male and female populations separately significance was lost and only a tendency was observed. According to sex-unstratified adjusted analysis, the *F* allele was associated with increased risk in patients having discopathies and/or osteochondrosis concomitant with herniation (Subgroup 2), all patients having discopathies and also in all patients having discopathies and/or osteochondrosis. All the associations were confirmed in males, while in females the *F* allele was a risk factor in crude analysis only for Subgroup 2.

Regarding patients with stenosis and/or spondylolisthesis, sex-unstratified analysis found no significant associations or tendencies. It is interesting to note, however, that in the gender analysis, in females FokI polymorphism showed an opposite tendency; the *F* allele seemed to be protective, while the *f* allele seemed to be associated and the *ff* genotype showed a risky tendency (*p* = 0.064).

The wild-type *VDR* *F* allele is considered to produce a more transcriptionally active receptor protein than the *f* allele [4,5,6]. Consequently, *FF* homozigosity should enhance vitamin D final effects and, thus, favor discopathies and the severe progression of discopathy and/or osteochondrosis to herniation. On the contrary, a reduced transcriptional activity of VDR, which is likely to occur in *ff* homozygous subjects, seems to favor stenosis and/or spondylolisthesis, especially in women.

Of note, a study performed in a geographically isolated population of both pre- and postmenopausal Italian women of the island of Lampedusa (located between the African coast and Sicily), showed that the *ff* genotype was associated with lower lumbar spine bone mass in comparison with *Ff* and *FF* genotypes [28].

Interestingly, a very recent study performed in 140 Iranian subject with diabetes evidenced that the *VDR* *ff* genotype was associated to lower vitamin D levels and this homozigosity may be regarded as “low responders” to vitamin D intake [29].

Several lines of evidence indicate that vitamin D homeostasis differs between men and women. Interestingly, it was found that women have lower levels of circulating vitamin D than men [30], and this effect was also observed in the Italian population [31]. However, it is not clear if this depends mainly on external factors or if the gender difference could be related to a differential vitamin D homeostasis in the two sexes [29,32]. It is tempting to speculate that a nutrigenic approach based on specific genotypes may be needed to protect patients with specific lumbar spine disorders, with particular attention to the gender-related differences.

A limitation of our study is the sample size. Stratification by gender and by pathological Subgroups reduced the number of subjects compared. An increase in the number of male and female cases and gender-matched controls will be necessary to strengthen statistical power and substantiate gender-related differences. Particularly, an increase in the number of subjects with stenosis and/or spondylolisthesis appears important to confirm if the observed opposite trend in term of the type of FokI allele associated to risk in these diseases is due to specific pathological features and/or is strictly gender-related.

In the future, it could be interesting to perform tissue specific studies analyzing the interplay between the different *VDR* variants and its ligand to give a biological explanation of the associations observed in patients. Moreover the authors should evaluate whether a particular vitamin D status, circulating levels and/or polymorphisms of vitamin D binding protein (DBP), diet or medications for treatment of neuropathic pain could affect vitamin D metabolism, thus predisposing to the development or progression of spine pathologies.

In conclusion, we here report the novel gender-related association of the functional *VDR*-FokI polymorphism and of the concomitant conventional risk factors with specific spine pathologies in the Italian white population.

In our opinion, the observation of pronounced gender differences could be crucial for the prospect of a personalized medicine approach and seems to strongly indicate the necessity of a gender-differentiated management of patients with lumbar spine disorders.

## 4. Experimental Section

### 4.1. Subjects and Clinical Assessment

Using a case-control design, 267 (149 males, 118 females) consecutive patients (hospitalized or outpatients) with lumbar spine disorders recruited for the European Genodisc Project and 254 (127 males, 127 females) asymptomatic controls (most were healthy volunteers, some were blood donors, few were subjects hospitalized for anterior cruciate ligament injuries or hallux valgus surgery) were enrolled at the I.R.C.C.S. Istituto Ortopedico Galeazzi (Milan, Italy). All subjects signed a written informed consent. Cases and controls were enrolled from May 2009 to December 2013. The study was approved by the Institutional Review Board ASL Città di Milano (Milano, Italy). The methods used in this study were in accordance with the Helsinki Declaration of 1975 as revised in 1996. The cohort of cases was the same of our previous published paper and inclusion/exclusion criteria for both cases and controls were previously reported [21]. However, in respect to our previous investigation the number of controls was increased including 21 more males and 13 more females, thus final number of controls (*n* = 254) comprised an equal number of males (*n* = 127) and females (*n* = 127).

Assessment of lumbar spine disorders and patient classification into subgroups based on detailed diagnosis were performed by an expert clinician in spine diseases by contrast-enhanced MRI 12 scans of the lumbar spine with a 1.5 T scanner (Avanto, Siemens, Erlangen, Germany) as described in our previous paper [21].

### 4.2. Conventional, Behavioral and Environmental Factors Evaluation

A questionnaire reporting the exposure to putative risk factors known from various studies as affecting the susceptibility to spine disorders [24] was obtained from each subject. The collected information included: family history (parents, brothers or sisters) of spine disorders, smoking habit, job physical demand for the majority of the working years (evaluated by the following score: 0 = sedentary; 1 = light; 2 = medium; 3 = heavy), hours/day spent driving or as a passenger in motorized vehicles (exposure to vibrations) and, finally, over the past year, the times a week spent in vigorous physical activity outside work involving twisting, bending or lifting, collectively indicated hereafter as leisure physical activity.

### 4.3. Determination of VDR-FokI Polymorphism

Blood samples were collected from the antecubital vein with evacuated ethylenediamine tetra acetic acid (EDTA) tubes (Vacutainer Tubes, Becton-Dickinson, Franklin Lakes, NJ, USA) from all the enrolled subjects. Genomic DNA was extracted according to the procedure of the DNeasy Midi kit (Qiagen, Duesseldorf, Germany). Polymerase chain reaction and restriction fragment length polymorphism (PCR-RFLP) methods were applied to detect the *VDR*-FokI polymorphism as previously reported [21].

Genotypes were designated by a lowercase letter (*f* allele, T nucleotide, mutated) for the presence of the restriction site and by a capital letter (*F* allele, C nucleotide, wild-type) for its absence.

### 4.4. Statistical Analysis

Kolmogorov-Smirnov test was used to assess the normal data distribution. Student’s *t*-test or Mann Whitney test were used to assess the differences between the frequency distributions of variables in cases and controls. ORs were calculated to set the association between specific categorical variables, alleles or genotypes and risk of spine pathologies in cases, controls, and subgroups of patients including males and females. Chi squared or Fisher’s exact test *p* values were reported as appropriate. Logistic regression was used to evaluate effects of confounders by obtaining adjusted ORs and 95% CIs for genotypes and alleles. Adjusted analysis included conventional risk factors: age, BMI, family history, smoke, physical job demand and exposure to vibrations. Leisure physical activity was not included as confounding because this kind of activity may derive both by personal sedentary habits and by absence of spine pain.

Significance level was held at 0.05. At variance, *p* values ≤0.10 were considered as a tendency. Statistical softwares used were GraphPad Prism version 5.00 (GraphPad software, La Jolla, CA, USA) and SPSS version 14.0 (SPSS Inc., Chicago, IL, USA).

## Supplementary Materials

Supplementary materials can be found at http://www.mdpi.com/1422-0067/16/02/3722/s1.
